# The Fifty-Fifth Annual Meeting—A. M. A.

**Published:** 1904-06

**Authors:** 


### The Fifty-fifth Annual Meeting—A. M. A.

ATLANTIC CITY possesses the advantage of being near Buffalo, thanks to the enterprise of the Lehigh Valley Railroad, hence a large number of physicians from this region is contemplating a visit to the greatest American seaside resort during the meeting of the American Medical Association to be held June 7-10, 1904. It is unnecessary to enlarge upon the attractions of Atlantic City which, at this season of the year is at its best, as its habitués well understand. It is a cosmopolitan resort, peopled summer and winter, by visitors from every quarter, and is not sustained by any coterie, or locality. Its sustenance is obtained from the wide, wide world.

Fortunate, indeed, is Buffalo in its location; it is only a night's ride from New York, Chicago, Philadelphia, Washington, Baltimore, Cincinnati, and Atlantic City. The latter is brought within easy reach of the Bison City by reason of Buffalo's excellent railway facilities in general; but particularly because the splendid Lehigh Valley company has made it so comfortable for the traveler to journey by that route; and, further, because its railroad traverses the most picturesque region east of the Allegany Mountains. A daytime ride through Central New York and its beautiful inland lake region; past the anthracite coal fields of Pennsylvania and over the mountain at Wilkesbarre; down the Mauch Chunk valley, skirting the Lehigh river to Bethlehem, where immense iron factories send skywards their tall chimneys, and then out into an agricultural landscape that is not excelled, furnishes a variety of scene that is at once an education and an entertainment.

The busy doctor needs just such a break in his strenuous routine as this trip affords, and it can be had with such a comparatively small outlay that he should think twice before rejecting such an opportunity. He will meet hundreds of his friends from every section of the country, and altogether spend a more delightful week than can be done at any other time during the season. By consulting the officials of the Lehigh Valley Railway at 369 Main street, Buffalo, full particulars may be obtained and special reservations will be made. Telephone, Seneca 2670.

The members, delegates and visitors to the Atlantic City meeting from Chicago will journey by the Grand Trunk and Lehigh Valley railways, according to an itinerary arranged by Dr. Liston H. Montgomery, of that city. The special train leaves Chicago at 11.05 a. m., Sunday, June 5, going through Illinois, Northern Indiana and Michigan by daylight, reaching Detroit at 8.05 p. m. The train will pass Buffalo in the early hours of Monday, and is scheduled to reach Philadelphia at 3.40, the same afternoon, and Atlantic City at 6.05, Monday evening. The liberality of the Lehigh Valley company in providing a luxurious train and an easy itinerary that enables the excursionists to view the most picturesque region in the world, is worthy of that great corporation and its courteous officials.

